# Toxic Shock Syndrome After Orthopaedic Surgery

**DOI:** 10.7759/cureus.42609

**Published:** 2023-07-28

**Authors:** Sharon M Fernandes, Amylene Luna, Thomas Hall, Brendan Madden

**Affiliations:** 1 Critical Care Medicine, St George’s University Hospitals NHS Foundation Trust, London, GBR; 2 Cardiothoracic and Intensive Care, St George’s University Hospitals NHS Foundation Trust, London, GBR

**Keywords:** toxic shock syndrome, emergency medicine resuscitation, general trauma surgery, bone and joint, medical critical care, orthopaedics surgery

## Abstract

Toxic shock syndrome (TSS) is a rare, life-threatening, acute multisystem disorder caused by exotoxin-producing streptococcal or staphylococcal bacteria. It is often characterised by pyrexia, diffuse erythroderma, malaise, confusion, and hypotension which may progress to multiorgan dysfunction and coma. A high index of suspicion along with immediate diagnosis and multidisciplinary management is required to improve the outcome of the disease.

A 62-year-old male presented to the hospital a week after an open reduction and internal fixation of a left wrist fracture. He was confused, febrile, and hypotensive with a generalised maculopapular rash on admission. Surgical wound sepsis was a top differential diagnosis; however, other possible sources were considered. Diagnostic imaging and echocardiography effectively ruled out other possible aetiologies. Despite fluids, vasopressor support, and appropriate antibiotics, he showed no significant clinical improvement. Admission blood cultures grew *Staphylococcus aureus* and after a multidisciplinary meeting, he was taken to the theatre for wound exploration, debridement, and removal of the metal plate. He was eventually weaned off vasopressor support and recovered well.

A high index of suspicion is important in recognising TSS in postoperative orthopaedic patients as wounds may appear healthy-looking and the onset of symptoms may be delayed. Early recognition, timely intervention, and multidisciplinary management are vital to the care of these patients.

## Introduction

Toxic shock syndrome (TSS) is a rare, life-threatening, acute multisystem disorder caused by exotoxin-producing streptococcal or staphylococcal bacteria. It is often characterised by pyrexia, diffuse erythroderma, malaise, confusion, and hypotension which may then progress to multiorgan dysfunction and coma. This classic presentation is rarely seen in patients with TSS complicating orthopaedic surgery [1]; hence, a high index of suspicion along with immediate multidisciplinary management is required to improve the outcome of the disease.

TSS is a rare but highly fatal cause of septic shock in postoperative patients. TSS as a complication of orthopaedic surgery was reported in 1984 where a total of nine cases were reported in orthopaedic patients. Patients presented at an average of 13 days postoperatively with a 27% case fatality rate [2]. The aetiology of TSS involves non-specific activation of T lymphocytes by exotoxins acting as superantigens leading to the release of cytokines. Staphylococci and streptococci pyogenes (Group A *Streptococcus*) are the two most common bacterial genera known to produce superantigens leading to TSS. Mortality from staphylococcal TSS is <3% whereas mortality for streptococcal TSS is 20-60% despite aggressive treatment [3]. The frequent delay in identification leads to poorer outcomes; hence, there is a need for greater clinical awareness in this patient group, and educating practice by increasing the body of case reports represents part of the solution.

## Case presentation

**Figure 1:**
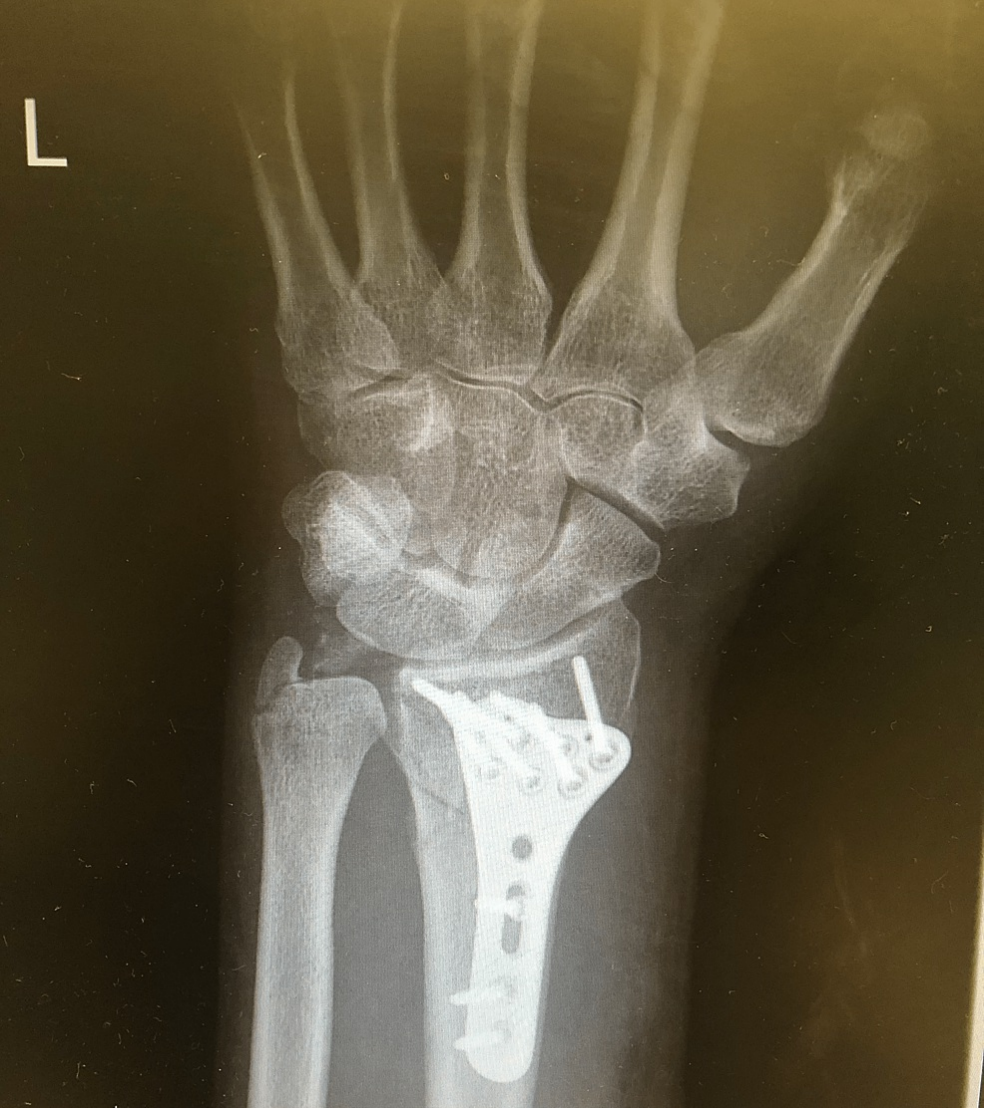
X-ray done after open reduction and internal fixation.

A 62-year-old male with no known comorbidities came to the hospital after a fall. An X-ray showed a closed left intra-articular distal radius fracture which required an open reduction and internal fixation (Figure 1). He was subsequently discharged home on the same day with a five-day course of co-amoxiclav as postoperative prophylaxis. A week later, he returned to the hospital with nausea, diarrhoea, confusion, and an erythematous rash.

On admission, the vital signs were as follows: systolic blood pressure of 70 mmHg, pulse rate of 130 beats per minute, respiratory rate of 30 breaths per minute, a temperature of 38.8°C and a Glasgow Coma Scale score of 14 (E4V4M6).

Significant findings on examination were a generalised blanching rash on the upper limbs and torso and a warm, mildly swollen, non-tender left wrist with an adequate range of movement and intact neurovascular status.

Admission blood tests showed a raised white cell count of 25.4, a raised C-reactive protein at 303, and a degree of mild renal impairment. Arterial blood gas analysis was consistent with metabolic acidosis with a raised lactate of 6.

Initial chest X-ray and CT scan of the head, chest, abdomen, and pelvis were normal. X-rays of the left wrist showed stable alignment of the implant with no signs of implant failure. The initial working diagnosis was wound sepsis and he was started on intravenous ceftriaxone, clindamycin, and Gentamicin per microbiology advice. Metronidazole was added later on.

He remained hypotensive despite fluid resuscitation and initiation of antimicrobial therapy prompting intensive treatment unit (ITU) admission for vasopressor support. He was reviewed by orthopaedics who deemed that the surgical wound is unlikely to be the source of infection. Bedside echocardiography was unremarkable, thereby ruling out endocarditis. Over the course of the patient’s ITU stay, the noradrenaline requirement continued to rise. Blood cultures grew *Staphylococcus aureus* and antibiotics were rationalised accordingly.

Over the succeeding days, he remained dependent on noradrenaline. A further wound review by orthopaedics showed mild wound dehiscence. A multidisciplinary meeting between ITU, microbiology, and orthopaedics was done and it was agreed that the patient should be taken to the theatre for wound exploration. Intraoperatively, bloody/turbid fluid (pus) was noted in the radial aspect superficial to the plate. Copious washout with debridement, curettage, and removal of metal work was subsequently performed.

The patient continued to improve postoperatively and was eventually weaned off inotropic support. He was transferred to the ward after 24 hours where he recovered well.

Differential diagnosis

In this patient who presented with fever, confusion, rash, and hypotension, the primary working diagnosis was septic shock. A wound infection post-open reduction and internal fixation is a top differential for the aetiology of sepsis; however, other possible sources were considered as well. Diagnostic imaging effectively ruled out endocarditis and a brain, chest, abdominal, and pelvic infection.

Outcome and follow-up

The patient improved significantly after surgical debridement and removal of the metal plate. Vasopressor was gradually weaned and he was later stepped down to the ward where he recovered well. Wound samples taken intraoperatively grew *Staphylococcus aureus* and gram-negative organisms (*Escherichia coli* and *Enterobacter cloacae*). Intravenous flucloxacillin and ertapenem were given for a total of two weeks. This was followed by a further four-week course of doxycycline, ciprofloxacin, and flucloxacillin. He was followed up at two, four, and six weeks and remained well with no further issues at four months.

## Discussion

TSS is a multiorgan dysfunction caused by toxins released by certain strains of bacteria. Exotoxins of *Staphylococcus aureus* and *Streptococcus pyogenes* are the most commonly implicated, with *Staphylococcus aureus* causing an annual incidence rate in the United Kingdom of 0.07/100,000 [4] and *Streptococcus* an annual incidence rate of 1.1 to 9.5 per million [5]. The mortality rate for TSS is around 5-15%. In one study, a fatality rate of up to 64% was noted in cases of streptococcal TSS in the United Kingdom. Recurrence of TSS is noted in 30-40% of cases. Previously, TSS was more often associated with the use of highly absorbent tampons during menstruation. A change in manufacturing and materials, however, caused a reduction in its incidence. On the other hand, there has been an increasing incidence of TSS in postoperative patients over the last three decades.

Although the exact mechanism of TSS has not been completely understood, it is believed to be an interplay between host immunity and the effect of exotoxins acting as superantigens. These superantigens have been identified to trigger excessive T-cell activation subsequently causing a cytokine storm.

Orthopaedic procedures have not been considered a risk factor for TSS and there have been very few reports of TSS complicating orthopaedic surgery. The first reported case of fatal TSS in an orthopaedic patient was published in 1984 [6]. An 18-year-old male came in for elective arthrodesis of the wrist and removal of an internal fixation device from the femur. He started having fevers on postoperative day one with negative cultures. The fever persisted over the succeeding days; however, no probable source of infection was identified. By day seven, he developed severe pyrexia of 41°C with diarrhoea, vomiting, and a desquamating rash. The surgical wounds remain benign-looking, but a wound swab from the wrist wound revealed rare gram-positive cocci in pairs. Antibiotics were escalated to penicillin, gentamicin, nafcillin, and clindamycin. The surgical wounds were subsequently explored and debrided. The patient, however, continued to deteriorate, eventually developing congestive heart failure, renal failure, and disseminated intravascular coagulation. Despite appropriate interventions, he suffered two cardiac arrests and attempts to resuscitate him 11 days postoperatively were unsuccessful.

Another case reported involved a healthy five-year-old girl who sustained a lateral condylar fracture of the humerus after a fall and underwent open reduction and K wiring of the lateral condyle under general anaesthesia [1]. The procedure was uneventful, with no issues on clinical review a week later. However, she presented to A&E two weeks after surgery with diarrhoea and vomiting, fever, and a rash. She was noted to be hypotensive during this time, febrile at 39°C with swelling and a serous discharge from the operative site. She remained hypotensive despite fluid resuscitation and was transferred to the paediatric intensive care unit for inotropic support. She was started on cefotaxime and was taken back to the theatre for washout and removal of K-wires. Clindamycin and teicoplanin were added postoperatively. Initial blood cultures were negative, however, the swabs from the pin sites grew *Staphylococcus aureus*. She was eventually weaned off inotropic support three days after admission and recovered well.

These cases, combined with the one discussed in this report, describe delayed presentations of TSS with septic shock, requiring ITU admission and return to surgery. In short, TSS carries a significant degree of morbidity and mortality based on these reports and merits a higher degree of suspicion. There is a good body of evidence for timely antibiotics and source control [7] in these patients.

## Conclusions

A high index of suspicion is important in recognising TSS in postoperative orthopaedic patients as wounds may appear healthy-looking and the onset of symptoms may be delayed. Providing appropriate organ support, identification of infectious sources, and surgical management are critical in preventing the associated mortality.
